# Scoliosis in spinal muscular atrophy in the era of disease-modifying therapy: a scoping review

**DOI:** 10.1007/s10072-025-08155-1

**Published:** 2025-04-02

**Authors:** Martina Gnazzo, Giulia Pisanò, Benedetta Piccolo, Emanuela Claudia Turco, Susanna Esposito, Maria Carmela Pera

**Affiliations:** 1 Department of Biomedical, Metabolic and Neural Sciences, University of Modena and Reggio Emilia, Modena, Italy; 2 Child Neuropsychiatry Unit, Department of Medicine and Surgery, University of Parma, Parma, Italy; 3 Pediatric Clinic, Department of Medicine and Surgery, University Hospital, University of Parma, Parma, Italy

**Keywords:** Spinal muscular atrophy (SMA), Scoliosis, Disease-modifying therapies (DMTs), Surgical intervention, Spinal deformities, Cobb angle

## Abstract

Spinal muscular atrophy (SMA) frequently causes scoliosis (up to 90% of cases), due to weakened axial muscles impacting motor and respiratory function. While new SMA treatments improve motor function, their effect on scoliosis progression is unclear. This scoping review (2016-October 2024) analyzed literature from Pubmed, MEDLINE, EMBASE, and Scopus, focusing on studies of SMA, scoliosis, and treatment approaches. The aim of this work was to describe the clinical features and the possible therapeutic approaches of scoliosis in the “new population” of pharmacologically treated SMA patients. We included all types of SMA as well as all the approved disease modifying therapies (DMTs). The review found significant variability in scoliosis presentation and surgical intervention among different types of treated SMA patients. Early pharmacological treatment may slow scoliosis progression, particularly in Type II SMA. Interestingly, Type I SMA patients, who typically don’t develop scoliosis due to severe hypotonia, showed an increased scoliosis onset. Larger studies are needed to fully evaluate the impact of different treatments on scoliosis progression in SMA, especially in Type I SMA patients, to establish updated standards of care.

## Introduction

Spinal muscular atrophy (SMA) is a severe neuromuscular disorder caused by biallelic mutations in the SMN1 gene, leading to a spectrum of clinical severities (Types I–IV) based on age of onset and motor milestones achieved [1–4]. 

Characterized by progressive muscle weakness and atrophy, SMA often results in functional limitations and skeletal deformities, particularly scoliosis, which can significantly impact respiratory function and overall health [1, 5, 6]. The management of SMA typically involves a multidisciplinary team with treatment strategies tailored to each patient’s needs and disease severity [6–8]. 

Scoliosis is one of the most common and significant complications in type II and type III SMA, because the loss of lower motor neurons leads to muscle weakness, atrophy, and motor function impairment [9]. Scoliosis is a three-dimensional deformity of the spine characterized by a lateral curvature and vertebral rotation, which can occur at various ages and due to different causes [10]. Scoliosis can lead to postural and respiratory dysfunctions, and in severe cases, result in significant physical limitations. The severity of scoliosis depends on the curvature angle, the type of curve, and the involvement of other structures, such as the ribs [11, 12]. 

Traditionally, scoliosis in SMA has been managed with bracing or surgery, based on curve severity and functional impact. Surgery is advised for skeletally immature patients with curves over 50 degrees or those with worsening function [8, 13]. Given its effects on respiration and mobility, early intervention is essential, especially in type I and II SMA [12–14]. 

The main surgical interventions are: posterior spinal fusion (PSF), growing rods (GRs, Magnetic or Titanium) and vertical expandable prosthetic titanium rib (VEPTR). The last two interventions are an alternative to early PSF because they are fusionless scoliosis surgery (FSS), allows for continued growth in height, and helps prevent thoracic deviation, potentially allowing for increased lung volumes over time [15–20]. 

The three relatively newly available disease-modifying therapies - nusinersen, onasemnogene abeparvovec, and risdiplam - have radically changed and improved patients’ outcomes [9, 21–28]. These current therapeutic approaches have led to significant improvements in outcomes, altering the natural history progression and resulting in the emergence of new phenotypes, characterized by a different progression of symptoms and a higher survival rate [29, 30]. 

Patients have achieved unprecedented motor milestones, such as sitting, standing, and walking with support [31]. 

The focus of most research has been on the motor and respiratory outcomes of SMA patients, with limited attention given to the scoliosis progression and management of scoliosis in individuals with SMA. This review aims to explore recent findings regarding scoliosis progression in treated SMA patients, including correlations between clinical features and different treatment approaches to scoliosis.

## Method

The scoping review was selected as the study design because of the exploratory nature of the research question. It followed the Joanna Briggs Institute (JBI) methodology as outlined in the online JBI reviewer’s manual for scoping reviews, with results adhering to the Preferred Reporting Items for Systematic Reviews and Meta-Analyses extension for Scoping Reviews (PRISMA-ScR) checklist.

This review is subdivided in two parts. In the first part (Part A) we analyze all articles published from 2016, the year when the first DMT for SMA was approved, to October 2024, focusing on the onset and progression of scoliosis in all types of treated SMA patients. Recent advances in treatment options in fact may have changed both the onset of scoliosis and the progression in children with SMA. In the second part (Part B) we examine literature (2016–October 2024) focusing on the various types of surgical intervention on scoliosis in SMA patients treated with DMTs. The rehabilitation program or the use of braces was not considered in the analysis of the various studies, as the aim was focused on surgical interventions.

### Inclusion and exclusion criteria

The article selection for the review adhered to the following inclusion criteria: patients with 5q SMA (hereafter referred to simply as SMA) described singularly or in comparison with different SMA subtypes, documented surgical techniques for scoliosis, reported scoliosis measurements (e.g., Cobb angle), and DMT treatment were applied to titles and abstracts (English-language, peer-reviewed journals), resulting in the selection of eight articles (part A) and ten articles (part B) (prospective and retrospective studies, case series, and RCTs) for in-depth analysis. Exclusion criteria encompassed articles where SMA patients served as a control group against other neuromuscular conditions, review studies, editorials, systematic reviews and meta-analyses.

### Search strategy

A systematic search was conducted on PubMed, MEDLINE, EMBASE, and Scopus using the following search criteria “[spinal muscular atrophy AND scoliosis],” published between 2016 and 2024, for part A. Instead, for part B, the search criteria were “[spinal muscular atrophy AND scoliosis AND scoliosis surgery]”, “[SMA AND arthrodesis],” “[magnetically controlled growing rods AND SMA].”

### Selection of the studies

During the screening phase, articles were screened based on their titles and abstracts, adhering to the predefined criteria. At this stage, articles without pertinent information on the subject, including those not in English, reviews, conference abstracts, editorials, viewpoints, conference proceedings and unpublished studies, were excluded. were excluded. Full texts of the articles derived from the screening phase were reviewed to determine whether they met the selection criteria. These full texts were also searched manually, which involved checking the reference lists of included papers to identify additional studies. Articles that were incomplete and those that did not meet the objectives were excluded. Two authors (MG and GP) independently reviewed the articles, determining their eligibility for inclusion, with any disagreements resolved by the supervisor (MCP).

### Data extraction

For part A, we have extracted and checked data on the characteristics of the studies. The following were extracted: title, author(s), years, type of SMA, % of scoliosis, n° of patients, age, Cobb angle (a measure of spinal curvature severity), treatment, and outcome of treatment on scoliosis. Note that the Cobb angle data may be represented as median, interquartile range (IQR), and range, depending on how it’s presented in the original studies. Additionally, not all articles in Table 1 focus primarily on scoliosis; in some cases, scoliosis is addressed as a secondary aspect of the study.

**Table 1:** Part A

Title	Author (s)	Year	Type of SMA	% of scoliosis	*N*° patients	Age (mean)	Cobb angle	Treatment	Outcome of treatment on scoliosis
Diagnosis and Management of Spinal Muscular Atrophy: Part 1: Recommendations for Diagnosis, Rehabilitation, Orthopedic, and Nutritional Care	Mercuri et al.	2018	1, 2 and 3	60–90%	NA	NA	20° in SMA Types 1 and 2, with surgical consideration at angles ≥ 50°	Multidisciplinary approach including physical therapy, respiratory support, nutritional management, spinal bracing, and surgical intervention (e.g., spinal fusion for severe cases)​	The findings suggest a high prevalence of early-onset kyphosis and scoliosis in children with severe SMA who receive onasemnogene abeparvovec gene therapy.
Long-term progression in type II spinal muscular atrophy: A retrospective observational study	Mercuri et al.	2019	2	NA	73	6y, 10 m	20° (requiring orthosis) and greater than 50° (requiring surgery)	NA	NA
Radiation dose reduction for CT-guided intrathecal nusinersen administration in adult patients with spinal muscular atrophy	Cordts et al.	2020	2 and 3	100%	13	30y, 2 m	NA	Nusinersen	This method allows for safer and more frequent monitoring and administration of nusinersen, which could indirectly benefit scoliosis management by optimizing SMA treatment.
Ultrasound-guided nusinersen administration for spinal muscular atrophy patients with severe scoliosis: an observational study	Zhang et al.	2021	2 and 3	100%	3	25y, 3 m	NA	Intrathecal administration of nusinersen using ultrasound-guided lumbar puncture	This study shows a safe and effective way to administer nusinersen in patients with severe scoliosis, but it doesn’t provide information on whether the nusinersen treatment had any impact on the scoliosis itself.
Treatment of spinal muscular atrophy with Onasemnogene Abeparvovec in Switzerland: a prospective observational case series study	Stettner et al.	2023	1 and 2	67% (SMA I)	9	5y, 2 m	NA	Onasemnogene Abeparvovec, also known as Zolgensma, administered in a single intravenous dose.	OA proved to be a potent treatment for SMA, resulting in notable motor improvements. However, the need for respiratory and nutritional support, as well as the development of scoliosis, highlights the importance of ongoing monitoring and support, particularly in SMA type 1 patients.
Early Development of Spinal Deformities in Children Severely Affected with Spinal Muscular Atrophy after Gene Therapy with Onasemnogene Abeparvovec—Preliminary Results	Soini et al.	2023	1 and 2	56%	16	1.54 ± 1.34 years	24 ± 27°, with kyphosis angles averaging 69 ± 15°	Onasemnogene abeparvovec gene therapy, with some patients also having prior nusinersen or risdiplam therapy	The study suggests that early-onset kyphosis is a notable clinical challenge in SMA children treated with onasemnogene abeparvovec gene therapy.
Spinal presentations in children with spinal muscular atrophy type 1 following gene therapy treatment with onasemnogene abeparvovec – The SMA REACH UK network experience	Wolfe et al.	2024	1	59%	NA	NA	NA	Onasemnogene abeparvovec (OA)	Presymptomatic treatment appears to significantly reduce scoliosis risk, while treatment after symptom onset may alter the natural course of the disease and the need for surgical intervention.
A horizontal and perpendicular interlaminar approach for intrathecal nusinersen injection in patients with spinal muscular atrophy and scoliosis: an observational study	Huang et al.	2024	2 and 3	40%	44	15.9 ± 7.3	39.9°	Intrathecal nusinersen injection using a novel ultrasound-assisted technique.	The treatment does not directly address scoliosis but facilitates the administration of nusinersen, which may help improve motor functions and quality of life in SMA patients. Improved muscle strength from nusinersen could potentially slow the progression of neuromuscular scoliosis in some patients.

NA: not available

Cobb angle: the Cobb angle data is given as the median, IQR, and range

All data are resumed in Table 1.

For part B, the data extracted were: study, n° of patients, type of SMA, surgical technique, mean age at surgery, Cobb angle pre/post/fu (°), pelvic obliquity pre/post/fu (°), thoracic kyphosis pre/post /fu (°), lumbar lordosis pre/post/fu (°), spinal length pre/post/fu (mm), respiratory outcome, motor outcome, follow-up, pharmacological treatment, and complications.

All data are resumed in Table 2.

**Table 2:** Part B

Study	*N*° patients	SMA type	Surgery technique	Mean age at surgery	Cobb angle pre/ post/ FU (°)	PO pre/ post/FU(°)	Thoracic kyphosis pre/ post / FU (°)	Lumbar lordosis pre/ post/ FU (°)	Spinal length pre/ post /FU	Respiratory outcome pre/ post	Motor outcome pre/ post	FU	Pharmacological treatment	Complications
Konigsberg et al. (2020)	8	-	PSIF modified to allow intrathecal drug administration	12.7 years	56.4°/21.1°/-	-	-	-	-	-	-	4 years	Post-operative Nusinersen	Not reported. 1 patient required revision surgery after 3.5 years
Lorenz et al. (2020)	17 2 had prior bilateral VEPTR	II	MCGR implanted parallel to the spine with rib-to-pelvis fixation	7.4 years	70°/29°/-	18°/5°/2°-7°	45°/34°/39°	Average values between 21°-31°	236 mm /289 mm/-	-	-	4.5 years	10 patients received Nusinersen during MCGR treatment	Implant dislocation (*n* = 1), rib fracture with implant dislocation (*n* = 3), wound healing problems (*n* = 2)
Machida et al. (2020)	4	-	PSF and additional L3 laminectomy	11.5 years	83°/37.8°/-	-	-	-	-	-	-	24 months	Treated with Nusinersen after 8 months	Aspiration pneumonia *n* = 1
Swarup et al. (2021)	66 15 treated with CWS 51 treated without CWS	II (68%), I (21%), III (5%)	MCGR (*n* = 17), VEPTR (*n* = 47), unknown (*n* = 2)	7.3 years	61°/47°/-	-	-	-	-	Requiring part-time/full-time respiratory assistance: 55%/45% Predicted FVC decreased by approximately 24% over an average of 5 years	-	2 years	42% of patients treated with Nusnersen over the course	Implant failure/migration (MCGR 23.5%, VEPTR 47%), surgical site infection (MCGR 12%, VEPTR 30%), pain (MCGR 6%, VEPTR 8.5%)
Drain et al. (2021)	1	I	MAGEC rods	18 months	67°/56°/-	-	-	-	-	Bi-pap dependent/-	-/sitting upright independently	5 years	Gene therapy	Wound dehiscence
Gaume et al. (2021)	59	I (*n* = 9), II (*n* = 47), III (*n* = 3)	Bipolar MIFLS	11 years	79.8°/39.2°/41.7°	24.1°/5.8°/5.9°	41°/26.4°/25°	36.4°/35.3°/37°	29.6 cm/34.6 cm/36.5 cm	FVC 0.53 L/0.67 L/-	Garchois brace *n* = 55/*n* = 11	5.2 years	26 Nusinersen	Mechanical (*n* = 8) or infectious (*n* = 5), unplanned surgeries (*n* = 6), implant removal (*n* = 1)
Kong Kam Wa et al. (2021)	4	I	Left GR (*n* = 1), bilateral spinal GR (*n* = 1), insertion of a spinal rod construction (*n* = 1), bilateral spinal rod insertion (*n* = 1)	4.75 years	-	-	-	-	-	-	-	-	Postoperative Nusinersen	Not reported
Matsumoto et al. (2021)	74	I (*n* = 14), II (*n* = 49), III (*n* = 2) Unknown *n* = 9 patients	VEPTR (*n* = 41), TGR (*n* = 12), MCGR (*n* = 15), Shilla GR (*n* = 2)	7.6 years	68.1°/41.8°/49.2°	-	-	-	-	FVC 63.9%/57.6% (1y fu) /56.3% (2y fu)		2 years	25 Nusinersen	Not reported
Xu et al. (2022)	1	III	PLF with a channel left on the vertebral laminae of L3-L4	16 years	63°/18%/-	9.5°/0.5°	-	-	-	FVC decreased/-	HFMSE 33/31, MFM 32/59, RULM 34/36	-	Nusinersen 30 days after surgery	Not reported
Cetik et al. (2023)	28	I	Dual MCGR (*n* = 15), unilateral MCGR (*n* = 4), VEPTR 8n = 3), self-distracting system (*n* = 5), TGR (*n* = 1)	61 months	78°/33°/33°	17°/4°/4°	70°/41°/40°	55°/42°/41°	238 mm/ 289 mm/ 304 mm	-		16 months	28 Nusinersen, 4 later switched to Risdiplam, other 4 switched to Onasemnogene Abeparvovec	Surgical site infection (*n* = 3), proximal anchor failures (*n* = 2), rod fracture (*n* = 1)

PO: pelvic obliquity; FU: follow-up; PSIF: posterior spinal instrumentation and fusion; VEPTR: vertical expandable prosthetic titanium rib; MCGR: magnetically controlled growing rods; PSF: posterior spinal fusion; MAGEC: magnetic expansion control; MIFLS: minimally invasive fusionless; GR: growing rods; TGR: traditional growth rods; PLF: posterolateral fusion; FVC: forced vital capacity, HFMSE: Hammersmith Functional Motor Scale Expanded; MFM: Motor Function Measure; RULM: Revised Upper Limb Module

The absence of data in some columns of Tables 1 and 2 indicates that the specific information wasn’t reported in the original source. Due to variations in study design and reporting methods across different publications, some data fields may be missing for specific studies.

### Assessment of the quality of studies and risk of bias from the review

The STROBE Checklist for cohort, case-control, and cross-sectional studies (combined) was used to rate the quality of studies included in this review [32]. 

Based on these criteria, the studies included in the review were judged to be of good, moderate, or poor quality.

The ROBINS-I tool (“Risk Of Bias In Non-randomised Studies - of Interventions”) was used to ascertain the risk of bias arising from the quality of included studies or the methods of this review [33]. 

To reduce the selection bias arising from included studies and the bias in rating the quality of studies, these procedures were initially carried out independently by two authors. Any discrepancies were resolved by joint consensus following the independent evaluations.

## Results

**Fig. 1:**
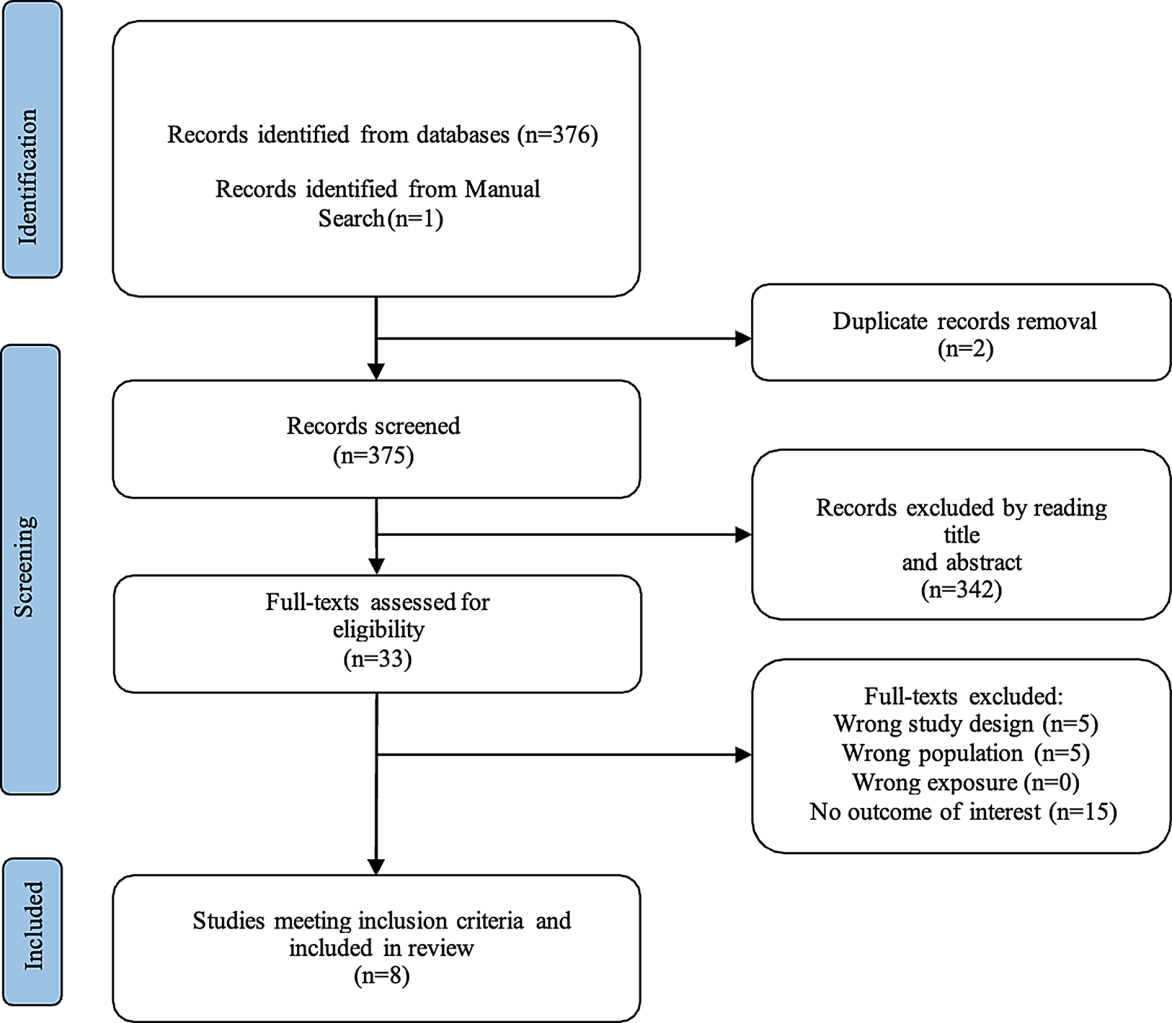
Flow-chart describing the Part A of the study selection process

**Fig. 2:**
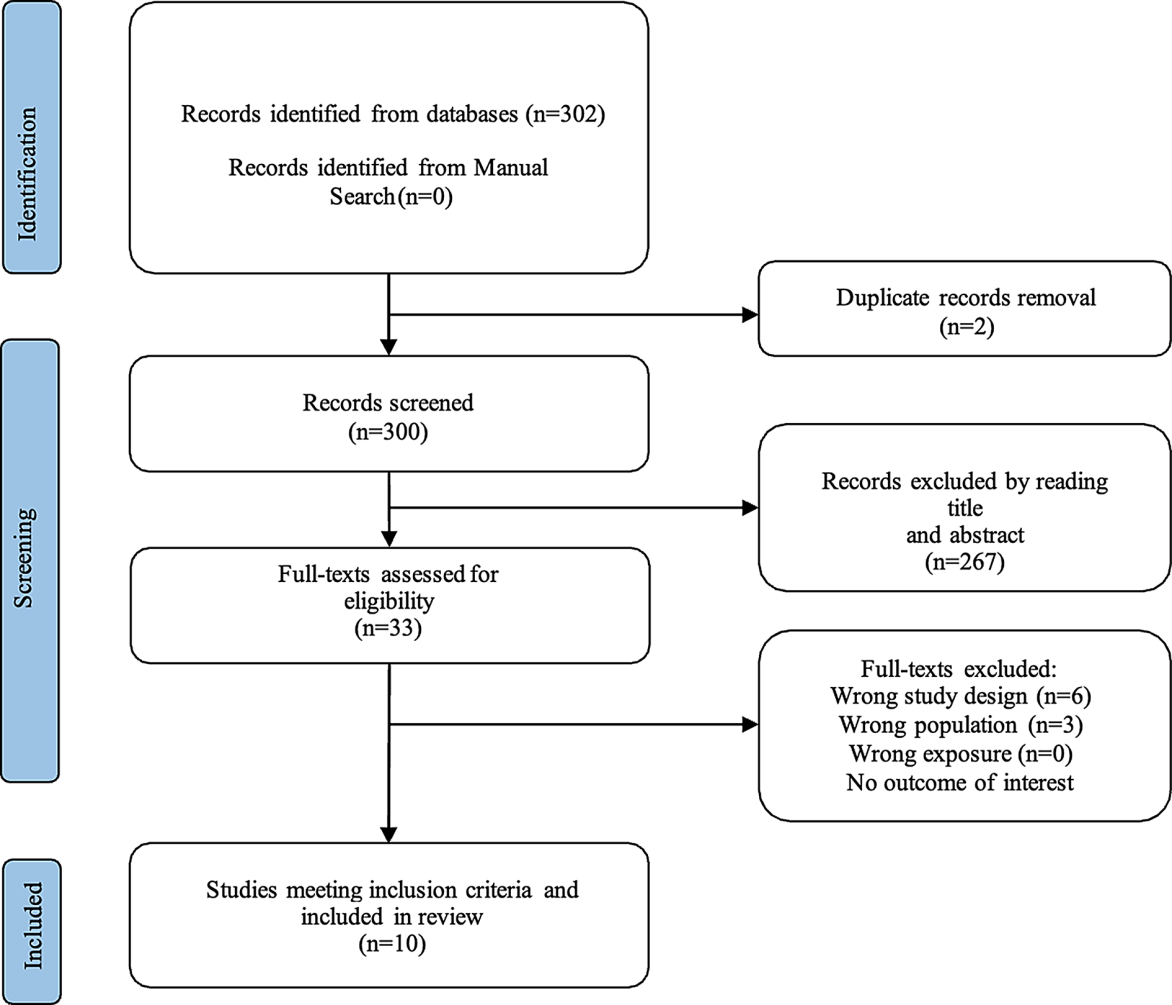
Flow-chart describing the Part B of the study selection process

Both the articles from Part A and Part B were selected and independently reviewed by two researchers (MG and GP). The articles retrieved from the search for Part A and Part B are summarized in the PRISMA Flow Chart in Figs. 1 and 2.

The Fig. 1 details the study selection process for Part A. The process started with 376 records identified from databases, plus one additional record from a manual search. After removing duplicates (2 records), 375 records were screened. Title and abstract review led to the exclusion of 342 records. Of the remaining 33 full-text articles assessed for eligibility, 25 were excluded (5 for wrong study design, 5 for wrong population, 0 for wrong exposure, and 15 for lacking the outcome of interest). Ultimately, 8 studies met the inclusion criteria and were included in the review.

In the Fig. 2, the process begins with identifying 302 records from databases. No additional records were identified through a manual search. After removing duplicates (2 records), 300 records were screened. Based on title and abstract review, 267 records were excluded. The remaining 33 full-text articles were assessed for eligibility, with 23 excluded due to various reasons: wrong study design (6), wrong population (3), no outcome of interest (14), and no other specified reason (0). Finally, 10 studies met all inclusion criteria and were included in the review.

### PART A

#### Prevalence of scoliosis by SMA type

Scoliosis is highly prevalent in SMA types 1, 2, and 3 with variable rates, depending on SMA type [34, 35]. 

SMA Type 2 exhibits the highest prevalence of scoliosis, with several studies reporting 100% in patients explicitly assessed for this condition. While SMA Type 3 has a less severe presentation, scoliosis remains a significant burden, especially as patients age [3]. 

#### Cobb angle and SMA type

Cobb angle measurements correlate with disease severity. In Type I SMA, early treatment is associated with smaller Cobb angles compared to treatment initiated post-symptom onset. However, severe scoliosis requiring surgery, with Cobb angles exceeding 50°, remains a challenge, particularly in Type II SMA patients [1, 36]. Type III patients generally exhibit more moderate Cobb angles, although progression may occur with age [37]. 

### Part B

#### Mean age at surgery and SMA type

The mean age at surgery for patients with Type I SMA varies significantly across studies, which could be due to several factors. Drain et al. (2021) reported a mean age of 18 months, which reflects the typically early onset and rapid progression of scoliosis in this group [38]. On the other hand, Konigsberg et al. (2020) reported a mean age of 12.7 years, which is much older. This discrepancy might be due to the differences in the cohorts studied [39]. 

In patients with Type II SMA, the results are more consistent. Both Lorenz et al. (2020) and Swarup et al. (2021) reported a mean age at surgery of around 7.4 years and 7.3 years, respectively [40, 41]. These aligned results indicate that for Type II SMA patients, surgery is typically performed at a relatively young age, as scoliosis develops progressively but remains severe enough to require intervention early on, often around the time when patients begin to lose the ability to sit independently [13]. 

For SMA Type III, Xu et al. (2022) reported a mean age of 16 years at surgery [42]. This is considerably older than the patients with Types I and II, reflecting the slower progression of scoliosis in Type III patients, who often retain more motor function and experience a more gradual onset of deformities. Surgery in these patients is generally performed later, as spinal deformities develop at a slower pace and may only become problematic as the patient ages [13]. 

#### SMA type and scoliosis surgery type

The choice of scoliosis surgery is closely related to the severity of scoliosis and the type of SMA.

Various spinal surgical techniques have been described for Type I SMA, including MCGR (magnetically controlled growing rods) and PSF (posterior spinal fusion). In patients undergoing PSF, modified techniques have been introduced to facilitate intrathecal nusinersen administration. This is necessary because the ossification following posterior spinal fusion restricts intervertebral access, making it difficult for the needle to reach the intrathecal space for nusinersen administration [42–47]. 

In SMA Types II and III, approaches such as VEPTR (vertical expandable prosthetic titanium rib) or TGR (titanium growing rods), with rib-to-pelvis fixation, are commonly employed in these patients, reflecting the different clinical challenges and stages of scoliosis [40, 48]. 

#### Surgery with major motor and respiratory outcomes

Scoliosis surgery has shown significant effects on both motor and respiratory outcomes, with certain surgical techniques contributing more significantly to functional improvement. Studies by Lorenz et al. (2020) and Gaume et al. (2021) found that MCGR and PSF led to improvements in motor function, including increased sitting ability and greater independence [40, 45]. 

While it is well recognized in clinical practice that some degree of worsening in motor function may occur immediately after surgery due to changes in posture and muscle recovery, this was only quantified in the study by Xu et al. (2022), where the Hammersmith Functional Motor Scale Expanded (HFMSE) temporarily decreased from 33 to 31, likely due to trunk muscle damage during surgery. Meanwhile, the Motor Function Measure (MFM) improved from 32 to 59, and the Revised Upper Limb Module (RULM) increased from 34 to 36 at the last follow-up [42]. 

These studies included patients with varying types of SMA and patients with moderate to severe scoliosis. However, the relationship between surgery and respiratory improvement is complex, and not all studies report consistent findings. For instance, in the study by Swarup et al. (2021), there was a reduction in the number of patients requiring respiratory assistance (from 55 to 45%), suggesting some improvement in respiratory function, although pulmonary function (measured by forced vital capacity, FVC) continued to decline over time [41]. In contrast, Matsumoto et al. (2021) reported a decline in respiratory function over time, suggesting that, despite surgery, patients with more severe scoliosis showed limited improvement in respiratory function [49]. The slight improvement observed in Swarup et al. (2021) may be attributed to the higher proportion of SMA type 3 patients in their sample, as these patients typically have milder disease and may respond better to surgery [13, 41]. 

#### Type of surgery and complications

The type of surgical approach chosen significantly influenced the incidence of complications. More invasive procedures, such as PSF and traditional growing rods (TGR), were associated with higher complication rates. Although Cetik et al. included only one TGR case in their cohort, they reported overall surgical site infections (11%), proximal anchor failures (7%), and rod fractures (4%), suggesting that mechanical and infectious complications remain concerns with this approach [50]. Similarly, Machida et al. reported aspiration pneumonia in 25% of cases following PSF with additional L3 laminectomy, emphasizing the respiratory risks linked to more invasive spinal fusion techniques [43]. 

Despite being designed to reduce surgical burden, less invasive growth-friendly techniques like magnetic controlled growing rods (MCGR) and vertical expandable prosthetic titanium rib (VEPTR) still presented notable risks. Swarup et al. reported that approximately 23% of MCGR cases experienced implant failure or migration, and 12% surgical site infections [41]. In comparison, VEPTR had higher complication rates overall, with 47% experiencing implant failure or migration and 30% having surgical site infections.

Lorenz et al., who investigated MCGR with rib-to-pelvis fixation, found implant dislocation in approximately 6% of cases, rib fractures with implant dislocation in 18%, and wound healing problems in 12%.^40^ Gaume et al. observed mechanical complications in approximately 14%, infectious complications in 8%, and unplanned surgeries in 10%, with 2% requiring implant removal [45]. Notably, Drain et al. documented wound dehiscence in their single reported case of magnetic expansion control (MAGEC) rods [38]. 

These findings underscore the variability in complication rates across different surgical approaches, reinforcing the need for careful risk stratification when selecting the optimal technique for each patient. However, differences in SMA type may also influence complication rates, as patients with SMA Type 1 tend to have more severe neuromuscular and respiratory impairments, potentially predisposing them to higher postoperative risks. Additionally, sample size disparities, surgical technique variations, postoperative care protocols, and patient-specific factors (e.g., nutritional status, comorbidities, and baseline respiratory function) could further impact complication outcomes.

While many studies either did not mention or only briefly referred to complications like implant failure (e.g., Swarup et al.), Kong Ka Wam et al. specifically addressed the concern of metal frame exposure, which may be a particular issue with permanent rods. [38, 39, 40, 41, 42, 43, 45, 49, 50, 51].

## Discussion

While disease-modifying therapies (DMTs) have shown improvements in motor function and survival, their effects on scoliosis remain unpredictable, with variability in outcomes depending on the timing of treatment and the specific SMA type [21, 49]. Scoliosis remains a major concern, affecting respiratory function, sitting balance, and overall quality of life [41]. 

The characteristic long C-shaped thoracolumbar curve arises from axial musculature weakness, with severity varying across SMA subtypes based on the degree of muscle weakness [13, 38, 42, 50]. 

Historically, SMA Type I patients did not develop scoliosis due to severe hypotonia and limited survival. However, with the advent of DMTs, their muscle strength, survival and motor function have dramatically improved [29]. These therapies have paradoxically increased the incidence of scoliosis in type I SMA where early and rapid progression significantly affects respiratory function and quality of life [1, 5, 50]. 

The persistence of scoliosis or the onset of kyphosis, particularly in treated type I SMA, shows the need for rehabilitation focusing on the trunk muscles and for the need for caution in repetitively trying to force the kids to sit without prior rehabiliation and strengthening of these muscles. The muscoloskeletal apparatus in SMA was not “planned” to sustain sitters or standers and more data is needed to understand the “best timing” to allow patients with SMA1 to sit.

On the other hand, early treatment with DMTs, especially pre-symptomatically, can delay onset or reduce scoliosis severity, particularly in milder SMA forms, yet scoliosis remains prevalent even in patients with improved motor function [34, 35]. Wolfe et al. (2024) found that presymptomatic treatment significantly reduces scoliosis risk compared to post-symptomatic treatment, underscoring the importance of early intervention [52]. Stettner et al., 2023 reported a prevalence of scoliosis in Type I SMA patients treated at 67%, suggesting that motor improvements do not fully mitigate spinal deformities [35]. 

In patients treated after symptom onset, there is often a phenotypic shift towards a less severe form, which influences the timing and need for surgical intervention. Particularly in Type II SMA, its onset tends to occur later, with varying degrees of progression depending on the severity of the underlying motor deficits [53]. 

Type III SMA patients typically present with less severe scoliosis, but progression can be observed over time, particularly as they age [52]. 

The 2018 standard of care for SMA, as outlined by Mercuri et al., provided a multidisciplinary framework for managing scoliosis. This included routine monitoring, the use of thoracolumbar orthoses to support spinal posture and growth-friendly instrumentation for younger patients with progressive curves exceeding 40–60° or compromising respiratory function. Definitive spinal fusion was recommended only after skeletal maturity to preserve growth potential, with the added guidance to ensure intrathecal access for therapies during surgical planning. These recommendations addressed a patient population managed without DMTs [1]. The current landscape necessitates updates to these guidelines. Advanced surgical techniques with minimum invasive intervention are now prioritized to preserve growth and minimize complications while maintaining spinal alignment [54, 55]. Modern strategies must also accommodate the requirements of intrathecal therapy and address the increased prevalence of scoliosis in longer-living SMA patients [56]. The variability in scoliosis progression across subtypes further underscores the need for individualized approaches, with faster progression observed in patients nearing surgical thresholds [57, 58]. Surgical interventions remain nuanced. Techniques like MCGR and posterior spinal fusion (PSF) have been associated with improved motor outcomes, such as better sitting balance and independence, but show variable impacts on respiratory function, particularly in severe, early-onset cases [23, 37, 40, 49]. Complications such as implant dislocations, wound healing issues, and rod fractures are more common with approaches like MCGR and PSF, whereas methods like VEPTR are associated with fewer complications but include challenges like rod fractures [50]. Early intervention remains critical to prevent severe deformities that can impair respiratory function and quality of life [51, 58, 59]. 

## Conclusion

Our review highlights the urgent need for a more standardized, evidence-based approach to the management of scoliosis in Spinal Muscular Atrophy (SMA). Comparing the 2018 recommendations with current needs highlights the evolving complexity of scoliosis management in SMA. Proactive monitoring and individualized interventions are essential to address legacy challenges while meeting emerging complications. The dynamic nature of SMA care underscores the urgency to refine and update consensus guidelines, ensuring optimal long-term outcomes for these patients [60]. 

Particularly as type I SMA patients live longer and achieve motor milestones, orthopedic and medical care must integrate advancements in DMTs and surgical techniques.

Moreover, surgical intervention should continue to play a central role in the care of patients with severe scoliosis, with careful selection of the most appropriate surgical techniques based on the patient’s SMA type and the progression of their disease [61]. Early and personalized surgical planning remains crucial in optimizing outcomes for these individuals.

In conclusion, scoliosis continues to be a major concern for individuals with SMA, and its management necessitates a comprehensive, multidisciplinary approach. This approach should integrate pharmacological treatments, surgical interventions and ongoing supportive care. The current landscape necessitates updates to standard of care guidelines, but there may be insufficient data to provide update on the best technique to be used, the best timing now with DMTs in place, and on outcomes, potential complications and safety in the long-term follow-up of treated SMA patients.

Future research should focus on refining treatment strategies, optimizing the timing of interventions, and exploring new approaches to further improve quality of life and functional outcomes for SMA patients [13]. 
